# Combined Alkaline Phosphatase and Phosphorus Levels as a Predictor of Mortality in Maintenance Hemodialysis Patients

**DOI:** 10.1097/MD.0000000000000106

**Published:** 2014-10-10

**Authors:** Jia-Feng Chang, Ying-Feng Feng, Yu-Sen Peng, Shih-Ping Hsu, Mei-Fen Pai, Hung-Yuan Chen, Hon-Yen Wu, Ju-Yeh Yang

**Affiliations:** Divison of Nephrology (J-FC, Y-SP, S-PH, M-FP, H-YC, H-YW, J-YY), Department of Internal Medicine, Far Eastern Memorial Hospital, New Taipei City; Graduate Institute of Basic Medicine (J-FC), Fu Jen Catholic University; and Department of Nursing (Y-FF), Jiate Excelsior Co, Ltd, Taipei, Taiwan.

## Abstract

Hyperphosphatemia-induced vascular calcification and higher alkaline phosphatase (ALP) levels-related high-turnover bone diseases are linked to mortality among patients with chronic kidney disease (CKD). Nonetheless, no large epidemiological study in patients with CKD has been conducted to investigate the interaction and joint effect of hyperphosphatemia and higher ALP levels on mortality.

We analyzed 11,912 maintenance hemodialysis patients from January 2005 to December 2010. Unadjusted and adjusted hazard ratios (aHRs) of death were calculated for different categories of serum phosphorus and ALP using the Cox regression model. The modification effect between serum phosphorus and ALP on mortality was determined using an interaction product term.

Both hypophosphatemia (<3.0 mg/dL) and hyperphosphatemia (>7.0 mg/dL) were associated with incremental risks of death (aHR: 1.25 [95% confidence intervals (CIs): 1.09–1.44], and 1.15 [95% CI: 1.01–1.31], respectively) compared to the lowest hazard ratio (HR) group (5 mg/dL ≤ phosphorus < 6 mg/dL). ALP levels were linearly associated with incremental risks for death (aHR: 1.58 [95% CI: 1.41–1.76] for the category of ALP > 150 U/L). In the stratified analysis, patients with combined higher ALP (>150 U/L) and hyperphosphatemia (>7.0 mg/dL) had the greatest mortality risk (aHR: 2.25 [95% CI: 1.69–2.98] compared to the lowest HR group (ALP ≤ 60 U/L and 4 mg/dL ≤ phosphorus < 5 mg/dL). Although the effect of hyperphosphatemia on mortality seemed stronger in higher ALP levels, the interaction was not statistically significant (*P* = 0.22).

The association between serum phosphorus levels and mortality was not limited to higher ALP levels. Regardless of serum ALP levels, we may control serum phosphorus levels merely toward the normal range. While considering the joint effect of ALP and hyperphosphatemia on mortality, the optimal phosphorus range should be stricter.

## INTRODUCTION

Cardiovascular (CV) disease is the most common cause of death in patients with chronic kidney disease (CKD), and extraosseous vascular calcification is a proven risk factor.^1,2^ The risk of vascular calcification corresponds with the deterioration of renal function and is highest in end-stage renal disease (ESRD).^3,4^ The reasons why patients on ESRD are at particular risk for vascular calcification are complicated, including disturbance of mineral metabolism, calcium-based therapies, chronic inflammation in a uremic milieu, and the active process of osteogenesis in vascular smooth muscle cells. Among them, hyperphosphatemia promotes vascular calcification accompanied with the expression of alkaline phosphatase (ALP).^5^

ALP is an enzyme responsible for dephosphorylation that is particularly derived from bone and liver, instead of vascular tissue.^6^ Higher serum ALP levels are associated with increased mortality in the general population and in patients on maintenance hemodialysis (MHD).^7–9^ The underlying pathogenesis between serum ALP and mortality has been speculated to involve high-turnover bone diseases and vascular calcification.^5–7^ During intramembranous ossification of vascular smooth muscle cells, ALP expresses to eliminate the calcification inhibitor pyrophosphate and provides only local availability for vascular tissue.^5,6^ In other words, key determinants of higher circulating ALP levels are bone and liver. Higher ALP levels are mainly associated with non-CV mortality in dialysis patients within 6 months, which suggests the important impact of skeletal-related events.^9^ Thus, ALP has been proposed as a novel predictor for high-turnover bone diseases.^9–11^

Previous studies have shown a “U”-shaped correlation between all-cause mortality and serum levels of phosphorus in the MHD population.^12–15^ Hypophosphatemia is connected to poor nutrition intake.^16^ The recommended target ranges of phosphorus are not yet conclusive. The Kidney Disease Outcomes Quality Initiative (KDOQI) suggested that the serum phosphorus should be maintained between 3.5 and 5.5 mg/dL in CKD stage 5D.^17^ However, a cohort study of 7076 hemodialysis patients from 2002 to 2004 revealed that patients who attained 2003 KDOQI targets did not have better survival compared to those who did not attain these targets.^18^ As suggested by the guidelines from Kidney Disease: Improving Global Outcomes, a phosphorus level should be “toward” rather than “within” the normal range.^19^ Furthermore, the Dialysis Outcomes and Practice Patterns Study conducted in Japan demonstrated a higher cutoff value of 6.5 mg/dL.^20^

It is well known that phosphate binders lead to many adverse effects and economic impacts.^21^ Aggressive control to achieve serum phosphorus levels within relatively lower range may compromise the quality of life of patients and does not definitely improve patient’s survival based on the current evidence.^19^ The optimal target ranges of serum phosphorus might differ in various clinic settings, depending on other existing risk factors for vascular calcification. It is recommended that while lowering phosphorus levels for patients with CKD, all available CKD and mineral and bone disorders (MBD) assessments should be taken into account.^19^ Both serum ALP and phosphorus are crucial surrogate markers for CKD–MBD, yet there are few reports about the interaction between hyperphosphatemia and higher ALP levels on mortality in patients with MHD. Specifically, whether the effect of hyperphosphatemia on mortality could be mitigated by lower ALP levels has not yet been investigated. The objective of the current study is to evaluate the interaction and joint effect of hyperphosphatemia and higher ALP levels on long-term mortality in a large number of patients with MHD. We assumed that the association between serum phosphorus levels and mortality would differ among different categories of serum ALP and tried to find the optimal range of serum phosphorus. Thus, we investigated the modification effect between serum phosphorus and ALP on mortality using an interaction product term in the Cox proportional hazard model.

## METHODS

### Cohort

We analyzed 11,912 patients undergoing regular dialysis at the Far Eastern Memorial Hospital, New Taipei City, Taiwan, and 87 hemodialysis centers belonging to Enfield Medical Co, Ltd, New Taipei City, from January 2005 to December 2010. Among these patients, 9631 with complete laboratory reports requested by the Taiwan Society of Nephrology were labeled as stable status and were included in this analysis. We excluded 47 patients on MHD for <90 days, 6 patients aged younger than 18 years, and 64 patients expiring within 1 month after the recruitment.

### Outcome

We linked to the national registry of death file to ascertain the death date. Patients who survived beyond December 31, 2010, were censored.

### Variables

We collected demographic data of each patient, which included age, sex, diabetes, and hemodialysis vintage (time since initiation of hemodialysis). Laboratory variables included hematocrit, phosphorus, calcium, intact parathyroid hormone (iPTH), ALP, albumin, creatinine, predialysis blood urea nitrogen (BUN), normalized protein catabolic rate (nPCR), dialysis dose (Kt/V urea by Gotch’s method), alanine aminotransferase (ALT), glucose, uric acid, total cholesterol, triglyceride, and ferritin.

We adjusted serum calcium according to the following equation: adjusted calcium = measured calcium + [(4.0 − serum albumin in g/dL) × 0.8]. We stratified serum phosphorus into 6 categories in 1.0-mg/dL increments (<3.0 to ≥7.0 mg/dL), and circulating ALP into 5 categories in 30-U/L increments (≤60 to >150 U/L).

### Statistical Methods

We presented continuous variables as mean ± SD or median with interquartile range and categorical variables as proportions. We calculated Spearman rank correlation coefficients between covariates of interest. We applied Cox regression to model the probability of death. The proportional hazards assumption was checked by graphical methods.

An interaction occurs when the impact of a risk factor on outcome is changed by the value of a third variable, sometimes referred to as effect modification.^22^ We evaluated if the effect of phosphorus on mortality was modified by ALP through incorporating a multiplicative interaction term in the multivariate model, that is, adding a variable whose value is the product of phosphorus and ALP. A significant product term indicates that there is an interaction between phosphorus and ALP on the probability of death.

A *P* value <0.05 was considered statistically significant. We used SAS 9.1 (SAS Institute, Cary, NC) to conduct the statistical analyses. The study had been reviewed and approved by the Research Ethics Review Committee of the Far Eastern Memorial Hospital (FEMH-IRB-101052-E, V.01; May 25, 2012).

## RESULTS

### Characteristics of the Study Population

**FIGURE 1:**
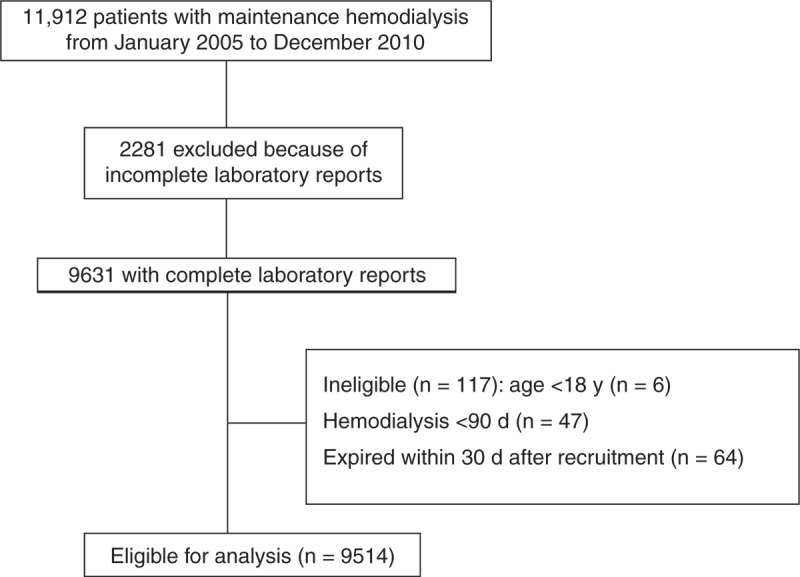
Flow diagram of patient enrollment.

The final study sample included 9514 patients with MHD (Figure 1). Baseline demographic characteristics and relevant laboratory data of the whole population of study subjects are summarized in Table 1. The mean age of the patients was 61.7 ± 13.4 years, and the median hemodialysis vintage was 22.4 (interquartile range: 8.1–60.4) months. Approximately, 46% of the study patients were male and 45.4% had diabetes. The median duration of follow-up from baseline laboratory measurements was 3.2 years. There were 3507 deaths during the 30,363 person-years of follow-up, corresponding to an annual mortality rate of 11.6%.

**TABLE 1:**
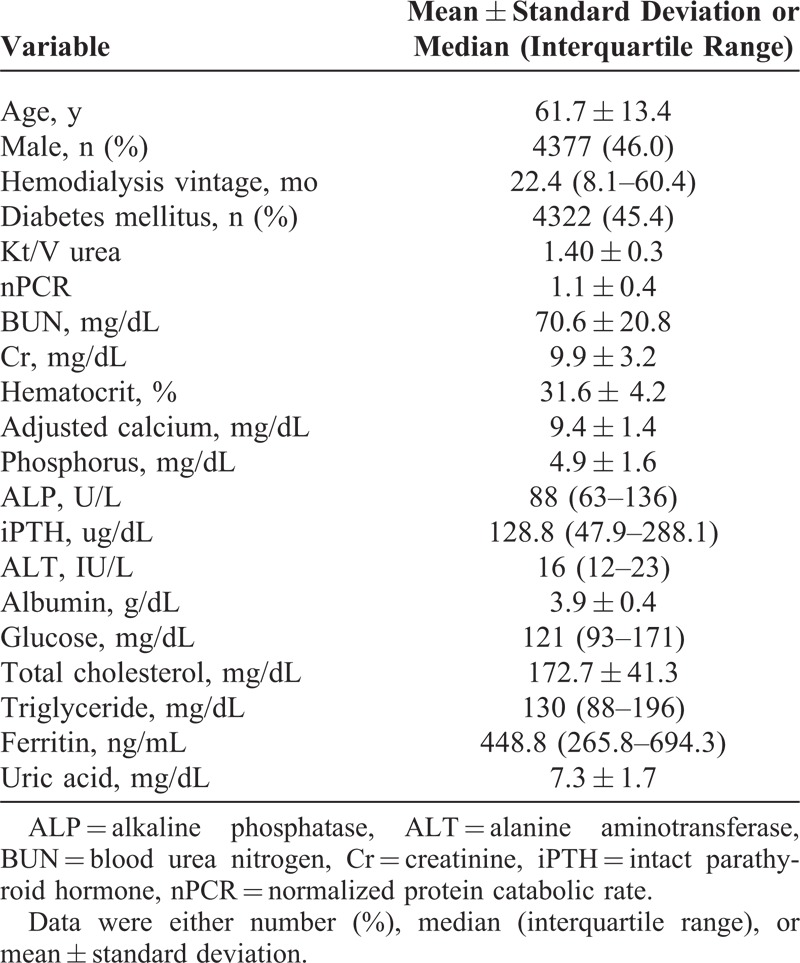
Baseline Demographic Characteristics and Relevant Laboratory Data of the Whole Study Cohort in 9514 Patients With MHD

Table 2 summarizes the bivariate correlation coefficients between serum phosphorus, ALP, and selected baseline variables in the 9514 patients with MHD. Age was inversely (*r* = −0.23) correlated with serum phosphorus concentration. Serum phosphorus was positively correlated with predialysis BUN (*r* = 0.39; *P* < 0.01), uric acid (*r* = 0.32; *P* < 0.01), serum creatinine (*r* = 0.33; *P* < 0.01), iPTH (*r* = 0.26; *P* < 0.01), nPCR (*r* = 0.25; *P* < 0.01), serum albumin (*r* = 0.19; *P* < 0.01), and total cholesterol (*r* = 0.14; *P* < 0.01). Serum ALP mainly correlated to iPTH (*r* = 0.22; *P* < 0.01). The correlations between serum ALP and other covariates were weak.

**TABLE 2:**
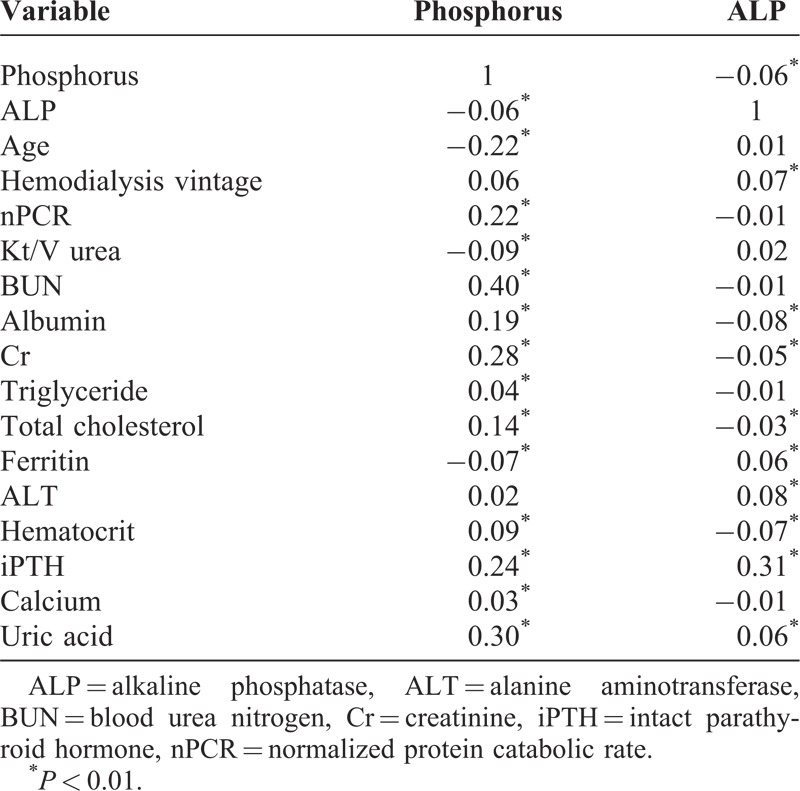
Correlation Coefficients Between Serum Phosphorus, ALP, and Various Variables

### Serum Phosphorus

**FIGURE 2:**
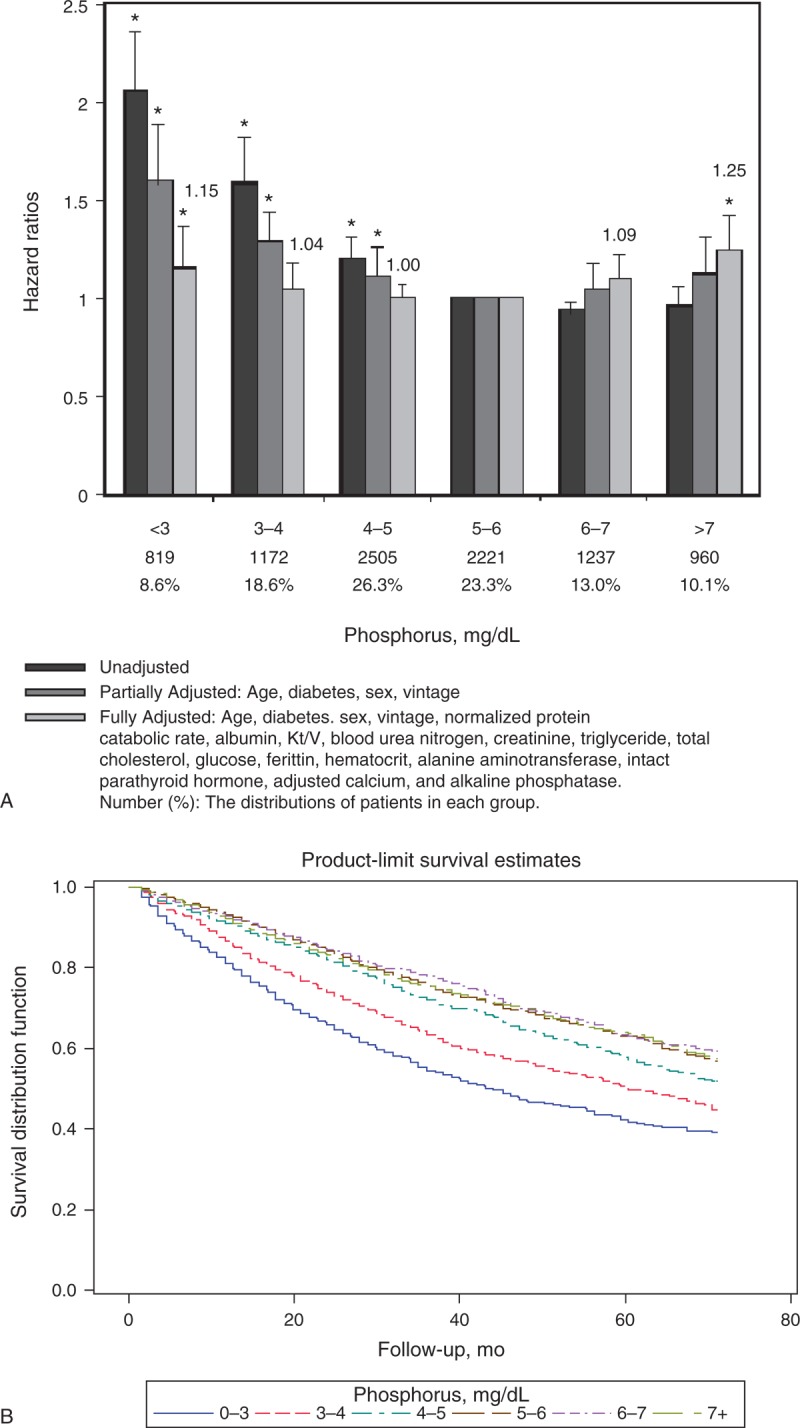
(A) Unadjusted, partially adjusted, and fully adjusted HRs of all-cause mortality across different categories of serum phosphorus levels in 9514 patients MHD during 30,363 person-years of follow-up. The lowest HR group (5 mg/dL ≤ phosphorus < 6 mg/dL) serves as the reference group. Note that the association between hyperphosphatemia and mortality was not significant until multivariate adjustments were made. (B) Kaplan–Meier survival curves among patients with different categories of serum phosphorus levels. HR = hazard ratio, MHD = maintenance hemodialysis.

Figure 2A shows the hazard ratios (HRs) and 95% confidence intervals (CI) for death among patients with different categories of serum phosphorus, considering the lowest HR group (5 mg/dL ≤ phosphorus < 6 mg/dL) as reference. The unadjusted analysis demonstrates a significant increase in HR among patients with lower serum phosphorus concentrations. With partial adjustment for age, diabetes mellitus (DM), sex, and hemodialysis vintage, we observed an increase in the HR for death among patients with higher serum phosphorus concentrations. After fully adjusting for multivariables (age, DM, sex, hemodialysis vintage, nPCR, albumin, Kt/V, BUN, creatinine, triglyceride, total cholesterol, glucose, ferritin, hematocrit, ALP, ALT, iPTH, and adjusted calcium), the incremental risk linked to lower serum phosphorus was attenuated and the risk associated with higher serum phosphorus was accentuated. Higher serum phosphorus concentrations (≥7.0 mg/dL) were associated with an increase in fully adjusted HR (aHR): 1.25 (95% CI: 1.09–1.44). The pattern of serum phosphorus and death association was U-shaped after full adjustments. Figure 2B shows Kaplan–Meier survival curves among patients with different categories of serum phosphorus levels.

### Serum ALP

**FIGURE 3:**
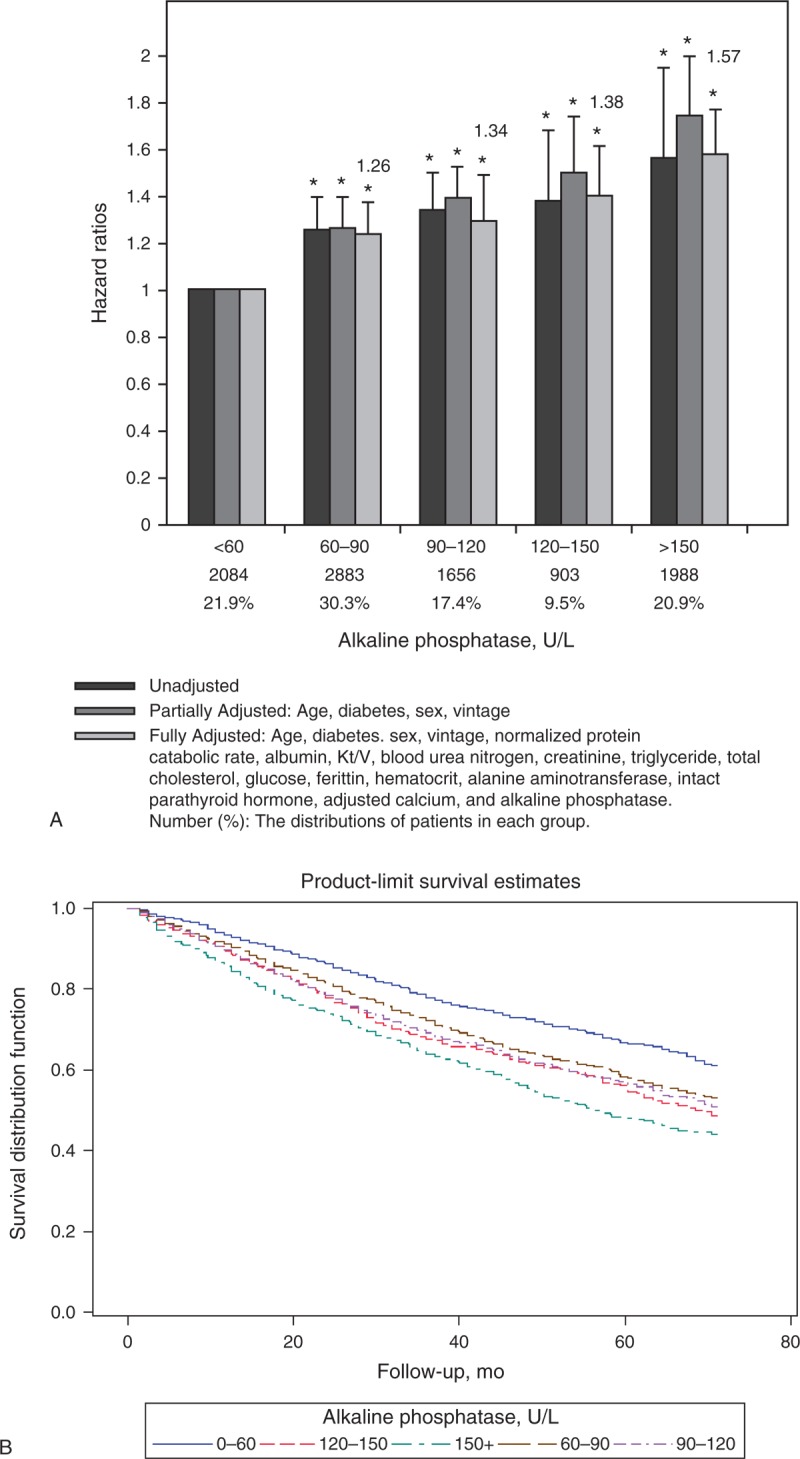
(A) Unadjusted, partially adjusted, and fully adjusted HRs of all-cause mortality across different categories of serum ALP levels in 9514 patients with MHD during 30,363 person-years of follow-up. Note that a linearly incremental risk of death associated with serum ALP in MHD patients is robust. (B) Kaplan–Meier survival curves among patients with different categories of serum ALP levels. ALP = alkaline phosphatase, HR = hazard ratio, MHD = maintenance hemodialysis.

Figure 3A shows the unadjusted, partially, and fully multivariable aHRs according to categories of serum ALP levels, using the lowest HR group (ALP concentration <60 U/L) as the reference. The unadjusted, partially, and fully multivariable-adjusted results all demonstrated a robust increase in HR among patients with higher serum ALP levels. Figure 3B shows Kaplan–Meier survival curves among patients with different categories of serum ALP levels.

### Serum Phosphorus and ALP

**FIGURE 4:**
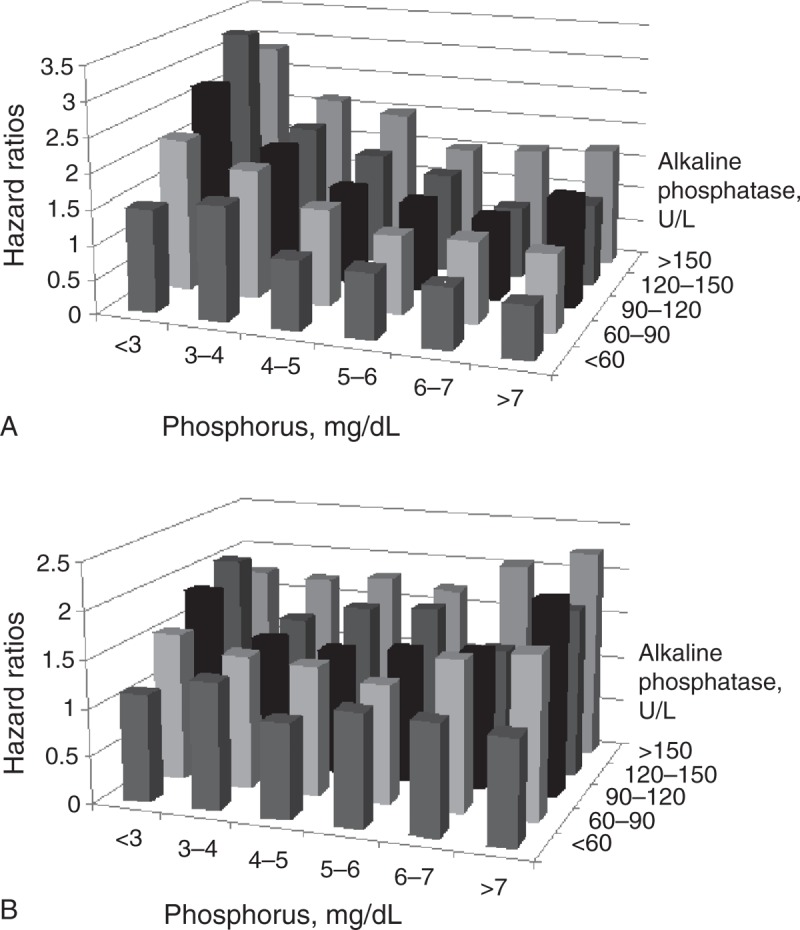
(A) Unadjusted and (B) fully adjusted HRs of all-cause mortality across the different categories of serum phosphorus and ALP levels in 9514 patients with MHD during 30,363 person-years of follow-up. The reference group (ALP ≤ 60 U/L and 4 mg/dL ≤ phosphorus < 5 mg/dL) has the lowest HR. Patients with higher ALP (>150 U/L) and hyperphosphatemia (>7.0 mg/dL) had the greatest mortality risk after full adjustments. Note that the association between serum phosphorus levels and mortality is not limited to higher ALP levels in the fully adjusted model. ALP = alkaline phosphatase, HR = hazard ratio, MHD = maintenance hemodialysis.

To visually represent the joint effect of 2 categorical variables on mortality, we used 3-D histogram to illustrate the unadjusted and fully adjusted HR of all-cause mortality across the different categories of serum phosphorus and ALP concentrations (Figure 4A and B). Patients with higher ALP (>150 U/L) and hyperphosphatemia (>7.0 mg/dL) had the greatest mortality risk (aHR: 2.25 [95% CI: 1.69–2.98]) compared with the lowest aHR group (ALP ≤ 60 U/L and 4 mg/dL ≤ phosphorus < 5 mg/dL). The unadjusted HR and fully aHR for death were apparently accentuated by higher ALP levels in both hyperphosphatemia (>7.0 mg/dL) and hypophosphatemia (<3.0 mg/dL) categories. Although the effect of hyperphosphatemia on mortality seemed stronger for higher ALP levels and less sensitive for lower ALP levels, the interaction was not significant in the model (*P* = 0.22). Hyperphosphatemia did not make the prediction more accurate for lower ALP levels. It also suggested that the effect of hyperphosphatemia on mortality was not limited to patients with higher ALP levels. While considering the joint effect of serum phosphorus and ALP, the optimal range indicated 4 mg/dL ≤ phosphorus < 5 mg/dL and ALP ≤ 60 U/L in our study.

## DISCUSSION

### Phosphorus and Mortality

Given that phosphorus is the main substrate of mineralization, hyperphosphatemia is regarded as a risk factor for vascular calcification and CV events. However, in accordance with previous studies,^12–14^ the association between hyperphosphatemia and mortality was not significant until multivariate adjustments were made (Figure 2). Serum phosphorus levels were correlated with age and several nutrition markers (Table 2). Insofar, as younger patients tend to intake more phosphorus,^23^ this might explain how the association between serum phosphorus and mortality was confounded by age. Because hypophosphatemia has often been related to malnutrition,^16^ the impact of hypophosphatemia on mortality was attenuated after adjustment of nutrition markers.^24^ However, both hyperphosphatemia (≥7.0 mg/dL) and hypophosphatemia (<3.0 mg/dL) were still associated with increased risk of mortality, independent of age, markers of nutrition, dialysis clearance, anemia, minerals, and liver enzymes. The impact of serum phosphorus level on the mortality is bidirectional; thus, we should control the serum phosphorus level toward the normal range.

### ALP and Mortality

Higher serum ALP values were incrementally and independently associated with increased risk for death before and after multivariable adjustment (Figure 3). One possible link between serum ALP and death involves vascular calcification. ALP promotes vascular calcification^6,25^ and consequently contributes to CV events.^7–9^ On the contrary, serum levels of ALP derived from apoptosis of osteoblasts reflect high bone turnover or metastatic bone diseases.^26,27^ MBD-related bone fracture is associated with an increased risk of all-cause mortality in the dialysis population.^28–30^ Other speculated causes mediating ALP and death include inflammation and malignancy.^7,8^ In brief, the contributions of serum ALP on the mortality did not operate through a single mechanism. The overall utility of ALP is considered as a marker of bone turnover in patients with CKD–MBD.^11^

### Interaction and Joint Effect of ALP and Phosphorus on Mortality

Given that both serum phosphorus and ALP correlate with vascular calcification and mortality, we assumed that the risk for death among patients with hyperphosphatemia would be augmented among patients with higher ALP levels and abated among those with lower ALP levels. The patients with higher ALP (>150 U/L) and hyperphosphatemia (>7.0 mg/dL) had the greatest mortality risk. However, the effect of hyperphosphatemia on mortality was not only in patients with higher ALP level but also in patients with lower ALP level. (*P* = 0.22 for the interaction term.) Phosphorus handling and ALP-driven pathways do overlap, and the finding of no interaction effect does not negate the fact that they share pathways. There are several reasons why the risk for death among patients with hyperphosphatemia was not abated among those with lower ALP levels. First, the serum ALP may not adequately represent the activity of tissue-bound vascular ALP. Second, the serum ALP also had a positive correlation with iPTH (*r* = 0.22), which is the traditional marker of high-turnover bone diseases instead of vascular calcification. Third, higher serum levels of ALP were mainly associated with higher all-cause death rather than CV death,^7–9^ implying the existence of intricate pathogenic pathways other than vascular calcification. Thus, the serum level of ALP might be viewed better as a marker of bone-turnover disorders.^11^ Current results suggested that serum phosphorus and ALP should be measured together and taken into consideration. Regardless of serum ALP levels, we may control serum phosphorus levels merely toward the normal range. The range of serum phosphorus 3 to 6 mg/dL seems to be acceptable and compatible with current guidelines.^17,19^ While considering the joint effect of ALP and hyperphosphatemia on mortality, the optimal phosphorus range should be stricter to avoid skeletal and CV events.

To the best of our knowledge, this is the first clinical study to show that the target range of serum phosphorus should be stricter while considering the joint effect of hyperphosphatemia-induced vascular calcification and higher ALP-related skeletal events. We analyzed a large cohort in Taiwan with detailed individual data and relatively long-term follow-up. The information of death obtained from the national registry of death files was also accurate. In terms of generalizability, such data provide a supporting evidence that the optimal range of serum phosphorus and ALP in Asia population is in accordance with other races.

Our study has several limitations. First, we failed to identify CV death from non-CV death, because the documented causes of death from national registry of death files were not adequately and consistently attributed. Second, our measured serum ALP was not a bone-specific type. The bone-specific ALP is measured with a more expensive immunoradiometric assay that is not widely available. Third, our conclusions are based on an observational cohort rather than a randomized controlled study. Fourth, the cross-sectional laboratory value might not reflect substantial intraindividual variability over time. Fifth, several important confounding factors, such as residual renal function, body mass index, or relevant medications, were not compulsory information requested by the Taiwan Society of Nephrology. Finally, the levels of other well-characterized circulating inhibitors of vascular calcification, such as fetuin-A and matrix Gla protein, were not collected in our database.

In conclusion, our study demonstrated the U-shaped association between serum phosphorus and mortality and a linear increment of risk of death associated with serum ALP. The association between serum phosphorus levels and mortality was not limited to higher ALP levels. Regardless of serum ALP levels, we should control serum phosphorus levels merely toward the normal range. While considering the joint effect of ALP and hyperphosphatemia on mortality, the target of serum phosphorus level should be stricter and within normal range.
